# Endoscopic Dilation with Bougies versus Balloon Dilation in Esophageal Benign Strictures: Systematic Review and Meta-Analysis

**DOI:** 10.1155/2018/5874870

**Published:** 2018-07-15

**Authors:** Iatagan R. Josino, Antônio C. Madruga-Neto, Igor B. Ribeiro, Hugo G. Guedes, Vitor O. Brunaldi, Diogo T. H. de Moura, Wanderley M. Bernardo, Eduardo G. H. de Moura

**Affiliations:** Gastrointestinal Endoscopy Unit, University of São Paulo Medical School, Dr. Arnaldo Av 455, São Paulo 01246-903, Brazil

## Abstract

**Background:**

The use of bougies and balloons to dilate benign esophageal strictures (BES) is a consolidated procedure. However, the amount of evidence available in scientific literature supporting which is the best technique is very low, despite the great prevalence and importance of such pathology. This systematic review with meta-analysis aims at comparing both techniques, providing good quality of evidence.

**Methods:**

We searched for randomized clinical trials (RCTs) published from insertion to November 2017, using MEDLINE, EMBASE, Cochrane Central Register of Controlled Trials, LILACS, and grey literature. After the data extraction, a meta-analysis was performed. The main outcomes were symptomatic relief and recurrence rate. The secondary outcomes were bleeding, perforation, and postprocedure pain.

**Results:**

We included 5 randomized clinical trials (RCTs), totalizing 461 patients. Among them, 151 were treated with bougie dilation and 225 underwent balloon dilation. Regarding symptomatic relief, recurrence, bleeding, and perforation rates, there were no differences between the methods. Concerning postprocedure pain, patients submitted to balloon dilation had less intense pain (RD 0.27, 95% IC −0.42 to −0.07, *P* = 0.007).

**Conclusion:**

We conclude that there is no difference between bougie and balloon dilation of BESs regarding symptomatic relief, recurrence rate at 12 months, bleeding, and perforation. Patients undergoing balloon dilation present less severe postprocedure pain.

## 1. Introduction

Benign esophageal strictures (BESs) are defined as any type of nonmalignant narrowing of the esophageal lumen. The physiopathological mechanisms of BESs are diverse and may entail embryonary defects, inflammatory injuries, and iatrogenesis. Also, there are several etiologic causes that may result from the combination of different mechanisms [1]. The most common clinical presentation is dysphagia, but others as retrosternal pain, regurgitation, and odynophagia might be present [1]. The impact on quality of life is usually remarkable since the patient frequently suffers from weight loss and aspiration pneumonia [2].

Modern bougies are made of polyvinyl chloride. The dilation procedure consists in passing a guidewire through the narrowing of the esophagus (fluoroscopy-guided or not) followed by bougination with gradually thicker dilators. This process generates an axial pressure on the stricture ring and theoretically poses a higher risk of esophageal perforation compared to the balloon dilation [3].

Balloon dilatation catheters were traditionally designed to treat patients with achalasia. In the early 1980s, they were introduced for the treatment of BESs. To dilate, the balloon should stand exactly at the stricture, in most cases secured by a guidewire. Then, it applies a radial pressure on the ring and ideally carries a lower risk of perforation. Other hypothetical advantages of the balloons are a greater precision (since it dilates only the exact narrowing) and the possibility to visualize the dilation process endoscopically as it occurs [4].

Despite all these theoretical advantages, there is no consensus in the available scientific literature to favor balloons over bougies [5, 6]. Also, there is no systematic review comparing both methods in patients with BES. Therefore, we developed this study to comprehensively search and compile all available data regarding this topic and ultimately provide practitioners with the most reliable evidence.

## 2. Objectives

The objective of this study is to compare the safety and efficacy of bougie and balloon endoscopic dilation in patients with BES.

## 3. Methods

This systematic review and meta-analysis was performed according to the PRISMA guidelines (Preferred Reporting Items for Systematic Reviews and Meta-Analyses) and was registered in PROSPERO (International Prospective Register of Systematic Reviews) under the registry number CRD42018085541.

## 4. Search

We searched MEDLINE, EMBASE, Cochrane Central Register of Controlled Trials, LILACS, and grey literature from inception to August 2017.

Our search strategy for MEDLINE (PubMed) was (Esophageal stenosis OR Esophageal Stenoses OR Esophageal Stricture) AND (Balloon OR dilatation OR savary OR bougie OR pneumodiltation OR pneumodilation). For other databases, we employed a simpler strategy: (Esophag^∗^ AND (Balloon OR dilatation OR savary OR savary-gilliard OR bougie OR bougienage OR pneumodilatation OR pneumodilation).

### 4.1. Inclusion Criteria/Eligibility

We considered a study eligible if it fulfilled the following criteria:

- Types of study: full-text randomized controlled trials (RCTs) with no language or publication date restrictions
- Types of participants: patients with benign esophageal strictures and no history of previous dilation
- Types of intervention: balloon and bougie dilation
- Types of outcomes:
  - Primary outcomes: symptomatic relief after dilation and recurrence rate at 12-month follow-up
  - Secondary outcomes: adverse event rates (bleeding, perforation and postprocedure pain)

### 4.2. Exclusion Criteria

- Patients with malignant esophageal strictures or previous esophageal dilation
- Studies that did not report the outcomes assessed in this review
- Studies with no extractable data

### 4.3. Study Selection

We combined studies identified in different databases and removed the duplicates. The first screening assessed titles and abstracts for eligibility. Then, a full-text analysis confirmed eligibility or excluded them from analysis.

In our meta-analysis, we included only studies providing complete data in the text, tables, or figures. If necessary, we assessed supplementary data available in online platforms (e.g., Clinical Trials or PROSPERO) or tried to contact the authors by email.

Study selection was performed by two independent researchers. In case of disagreement, it was resolved by consensus with a third researcher.

### 4.4. Methodology Quality and Risk of Bias Assessment

The studies were assessed using Cochrane's risk of bias tool [7], which classifies the risk of bias as low, high, or unclear.

### 4.5. Data Extraction

Two independent researchers extracted data using a standard Excel spreadsheet detailing the absolute numbers reported in the articles. We extracted data regarding the following dichotomous outcomes: symptomatic relief, recurrence, bleeding, perforation, and postprocedure pain.

### 4.6. Analysis

We used the RevMan software version 5.3 to perform all analyses and employed mean or absolute risk difference. We applied the Mantel-Haenszel test for categorical variables and considered statistically significant results with 95% confidence interval (CI) and *P* < 0.05.

We used the method of inverse variance and fixed effect model to provide the forest plots. Heterogeneity was assessed with the Higgins test [8](*I*^2^), and values higher than 50% were considered highly heterogeneous. Funnel plot analyses were also employed to assess publication bias across studies.

If *I* [2] was higher than 50%, we searched for outlier studies through the funnel plot. Articles outside the limits of the funnel were excluded, and heterogeneity was reassessed. If the sample became homogenous (*I*^2^ < 50%), the excluded studies were considered true outliers and were permanently removed. If we did not find an outlier, we considered true heterogeneity and switched from fixed to random effect analysis.

## 5. Results

**Figure 1:**
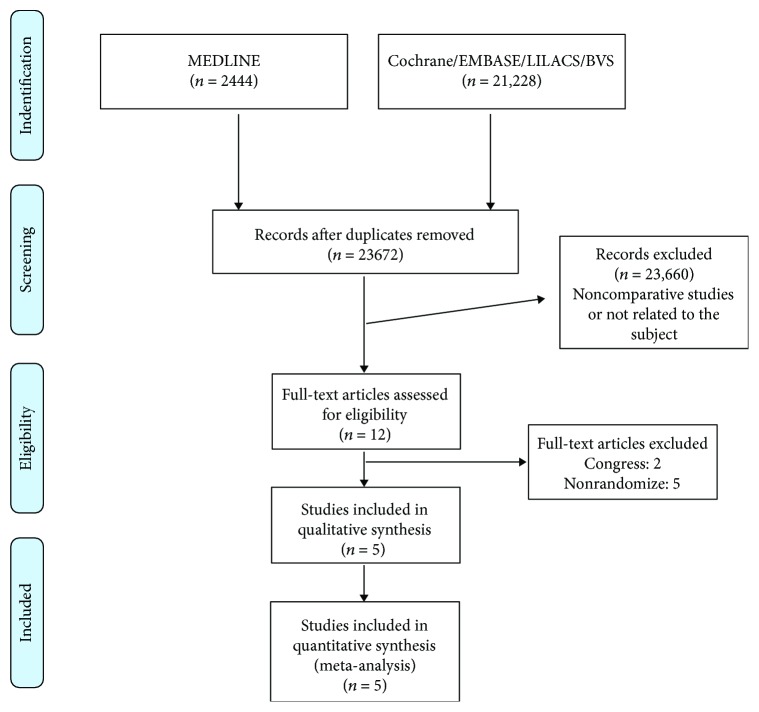
Flow diagram summarizing the selection process.

Initially, we identified 23,672 studies. After the title/abstract assessment, we selected 12 articles for full-text evaluation. Finally, 5 RCTs [9–13] fulfilled eligibility criteria and were included in our analysis.  Figure 1 summarizes the selection process.

The 5 RCTs included in our review enrolled a total of 461 patients, including 197 bougie and 264 balloon dilations.

The selected trials were developed in several countries from different continents and enrolled a great variety of patients and clinical presentations. However, all patients underwent esophageal dilation at least up to 15 mm.  Table 1 summarizes the studies' characteristics.

## 6. Risk of Bias

**Figure 2:**
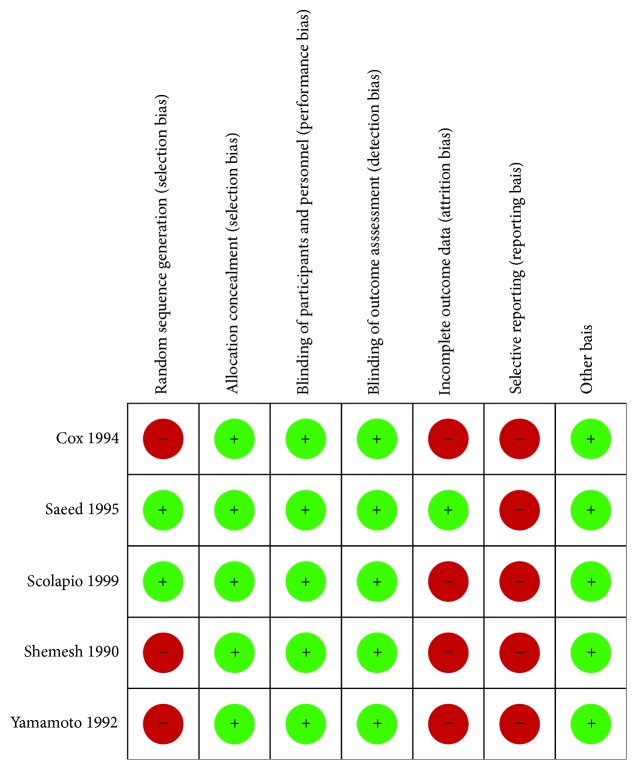
Risk of bias within studies.

**Figure 3:**
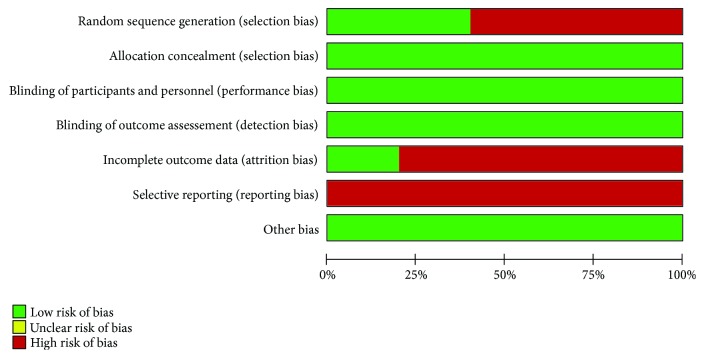
Risk of bias across studies.

Assessing risk of bias, we identified issues during randomization and blinding processes but adequate intention to treat analysis in most of the included trials. Therefore, we assigned fair quality to all studies. Figures 2 and 3 synthesize the risk of bias assessment.

## 7. Outcomes

### 7.1. Symptomatic Relief

**Figure 4:**
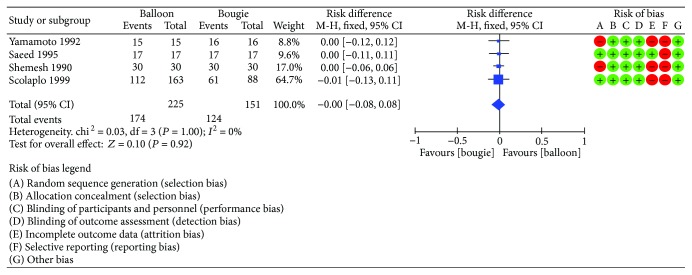
Symptomatic relief: forest plot.

Four studies [10–13] enrolling 376 patients were included. Among them, 151 underwent bougination while 225 underwent balloon dilation. Regarding postprocedure symptomatic relief, the meta-analysis found no difference between the groups with *I*^2^ = 0% (95% CI [−0.08, 0.08]).  Figure 4 shows the forest plot for symptomatic relief.

### 7.2. Recurrence Rate

**Figure 5:**
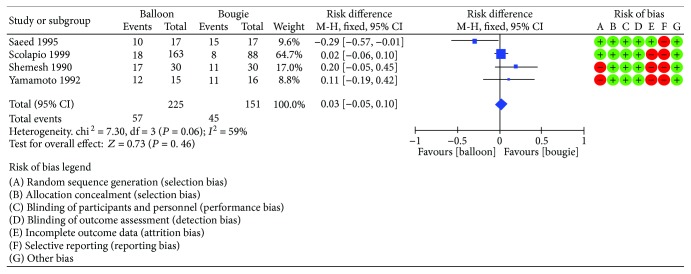
Recurrence rate: forest plot.

**Figure 6:**
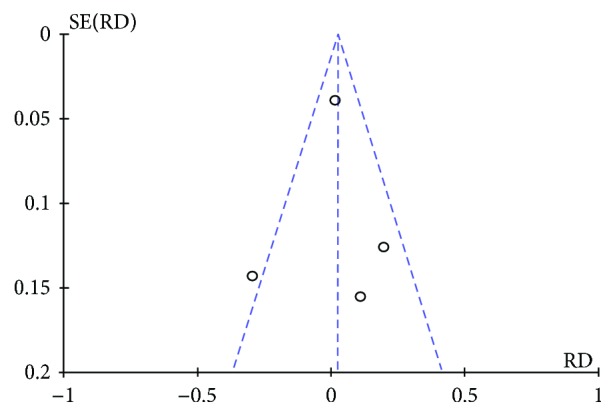
Recurrence rate: funnel plot demonstrating an outlier.

**Figure 7:**
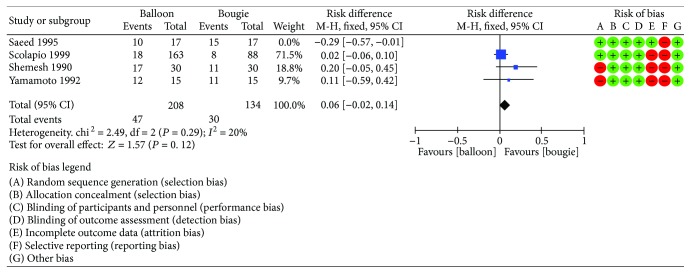
Recurrent rate: forest plot after outlier exclusion.

Four studies [10–13] reported the recurrence rate at 12 months. The risk difference was 0.03 (95% CI [−0.05, 0.10]) with *I*^2^ = 59% (Figure 5). After the funnel plot analysis, we identified and removed an outlier (Saeed et al.) (Figure 6). Then, we pooled data again and found a decrease in heterogeneity (*I*^2^ = 20), but still no difference between the methods (Figure 7).

### 7.3. Bleeding

**Figure 8:**
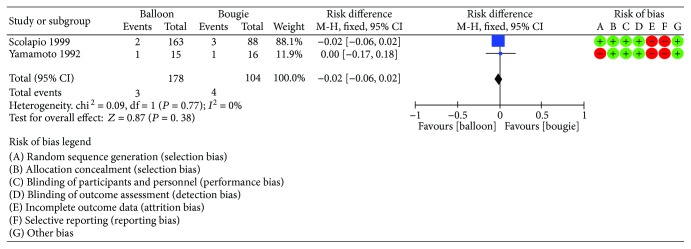
Bleeding: forest plot.

Two RCTs [11, 13] enrolling a total of 282 patients reported bleeding rates. Among them, 104 patients were allocated for the bougie group and 178 for the balloon group. This analysis was highly homogenous (*I*^2^ = 0%), and the risk difference was −0.02 (95% CI [−0.06, 0.02]). Therefore, we found equivalence of methods regarding bleeding rates (Figure 8).

### 7.4. Perforation

**Figure 9:**
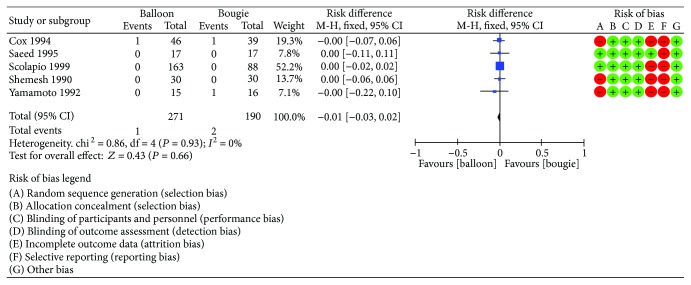
Perforation rate: forest plot.

For this analysis, we included five RCTs [9–13]. A total of 461 patients were enrolled, 190 treated with bougie and 271 with balloon dilation. Again, the meta-analysis identified highly homogenous data (*I*^2^ = 0%) with risk difference between groups of −0.01 (95% CI [−0.03, 0.02]). Therefore, we found no difference concerning perforation rates (Figure 9).

### 7.5. Postprocedure Pain

**Figure 10:**
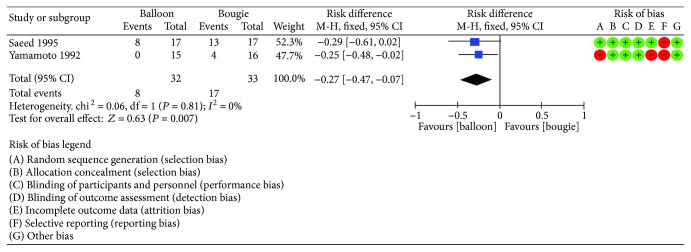
Postprocedure pain incidence: forest plot.

Two trials [10, 13] reported postprocedure pain incidence. A total of 65 patients were enrolled (33 in the bougie group and 32 in the balloon dilation group). This analysis was highly homogenous (*I*^2^ = 0%). The balloon group had a significantly lower incidence of pain after the procedure (RD 0.27, 95% CI 0.08–0.47, *P* = 0.007).  Figure 10 shows the forest plot regarding postprocedure pain incidence.

## 8. Discussion

Systematic reviews and meta-analyses are statistic tools used to pool data from different studies, aiming at improving the level of evidence available. Regarding the treatment of BESs, we identified the absence of high-quality randomized clinical trials, particularly in the last 20 years. Most published articles are case series, which are fairly reliable in terms of level of evidence. Therefore, this is the most trustworthy study available concerning the endoscopic treatment of BESs [14].

This systematic review included all 5 RCTs [9–13] available in the literature, which were all published before the 2000s. This fact demonstrates the lack of current evidence, which is necessary to guide treatment and management of such complex disease. Usually, the scientific community stops researching about a specific theme when steady data is found and we achieve a consensus. However, our systematic review clearly found conflicting results when comparing techniques, and therefore refuses any kind of consensus. In 2015, the World Society of Emergency Surgery published a consensus [15] observing that there was no clear advantage of any of the methods in peptic esophageal strictures, but Savary dilators would be more reliable and effective due to the possibility to “feel the resistance to dilation under the operators hands”. However, such statement was not supported by any good quality study, being rated as level 4 recommendation.

Our meta-analysis included 461 patients with different etiologies for the BESs, such as peptic, Schatzki ring, postradiation, postanastomotic, and caustic. Also, different kinds of bougies (e.g., Savary-Gilliard and Puestow) and balloons (e.g., CRE, Rigiflex, and Bard) were used for dilation in the studies. These factors probably explain the high heterogeneity found in the recurrence rate analysis. Particularly, Saeed et al. was the main cause for the high heterogeneity and therefore was excluded as an outlier. The remainder were homogenous, but the result still showed equivalence of methods.

The employment of different bougies, each with particular physicochemical characteristics, may also be a confounding factor. To date, no randomized clinical trial compared thermoplastic (Savary-Miller) to metallic (Eder-Puestow) bougies. In our personal experience, the metallic ones carry higher perforation rates while handling is more challenging. Thus, we no longer employ such dilator in our daily practice.

Similarly, there are no high-quality studies comparing high- to low-complacency balloons. Theoretically, the highly complacent ones pose a higher risk of perforation due to their capacity to mold according to the shape of the stricture. This mechanism results in overpressure beyond the narrowing, which might result in esophageal perforation. However, there is no concrete literature supporting this hypothesis.

Regarding the cause of BESs, the lack of literature is even more impressive. Some etiologies such as peptic present a better response to dilation because the inflammatory process is usually limited to superficial layers and spares the *muscularis propria* [16]. Moreover, the efficacy of PPI treatment decreased the number of patients suffering from peptic strictures. Controversially, other etiologies such as postradiation and caustic carry full-thickness inflammation and fibrosis. In these cases, the symptomatic relief after dilation is usually shorter [16]. Therefore, it is always imperative to consider not only the technique but also the etiology of the BES when analyzing outcomes.

Concerning the interval between sessions, some authors advise early redilation regardless of symptoms, while others recommend dilation according to the patient's complaints [17]. Again, there is no current consensus regarding this topic, and each trial included in our meta-analysis may have employed a different interval between sessions. This fact might be considered another confounding factor.

The consistency of the diet is also central. The included RCTs did not specify if the patients were kept on a liquid, soft, or solid diet. This information is essential since the failure of treatment should be determined based on symptoms, especially dysphagia and weight loss. It is not uncommon to find patients with severe strictures but only mild or none dysphagia. Those cases should not be deemed failure of treatment because the patient is able to eat the minimum nutrients and calories needed with an acceptable quality of life.

Considering all aforementioned limitations and confounding factors, our systematic review was the first to show that patients treated with bougie dilation suffered more frequent episodes of postprocedure pain than those treated with the balloon. In the meantime, we found no difference regarding symptomatic relief, recurrence rate, bleeding, and perforation. Progressive dilation sessions and particular orientation of diet possibly explain our results. Moreover, the short follow-up (12 months) may also have contributed to this result, since it is insufficient to adequately assess the response of complex and refractory esophageal strictures.

The postprocedure pain was more present in the bougie dilation group. The two trials included in this analysis did not mention which scales were used to measure the symptoms. Thus, we used this data as postprocedure pain incidence, regardless of its intensity. The endoscopic dilation with a TTS (through the scope) balloon allows the endoscopist to see the dilation process as it occurs. Opposingly, the bougie dilation is performed blindly or fluoroscopy-guided, theoretically increasing the risk of deep laceration and postprocedure pain. Another plausible explanation for this finding is that the bougie passes through the soft palate and crosses the upper esophageal sphincter, while the balloon is inflated in a distal position, after those structures. However, there is no literature supporting these theories.

In summary, the efficacy and complication rates of bougie and dilation are similar. Considering this level of evidence 1A study, we can state that there is no significant difference between methods to recommend one over the other. However, all the confounding factors and limitations should be considered. The difficult to perform good quality trials in this field is very clear, so we hope our study is able to guide and encourage the development of new randomized clinical trials, aiming to fulfill all the remaining gaps in the literature.

## 9. Conclusion

We conclude that there is no difference between bougie and balloon dilation of BESs regarding symptomatic relief, recurrence rate at 12 months, bleeding, and perforation. Patients undergoing balloon dilation experience less severe postprocedure pain. In addition, we have identified many confounding factors and limitations that should be addressed by specifically designed trials.

## Figures and Tables

**Table 1:** Characteristic of the included studies. Yamamoto et al. and Saeed et al. did not specify the etiology of the BESs.

Study	Control (*n*)	Intervention (*n*)	Population
Shemesh, 90	Savary-Gilliard (30) *Dilation with bougies up to 17 mm*	Microvasive (30) *Dilation with balloons up to 18 mm*	Peptic stricture (39) Caustic stricture (11) Postoperative (10)

Yamamoto, 92	Eder-Puestow (16) *Dilation with bougies up to 15 mm*	Medi-Tech (15) *Dilation with balloons up to 20 mm*	n/a

Cox, 94	Celestin + Eder-Puestow (39) *Dilation with Celestin bougies up to 18 mm, followed by Eder-Puestow bougies up to 19.3 mm*	Rigiflex Microvasive (46) *Dilation with balloons up to 20 mm*	Peptic stricture (61) Barrett's esophagus (8) Postoperative (11) Postesclerotherapy (1) Postcricoid (1) Systemic sclerosis (2) Caustic stricture (1)

Saeed, 95	Savary-Gilliard (17) *Dilation with bougies up to 15 mm*	Rigiflex Microvasive (17) *Dilation with balloons up to 15 mm*	n/a

Scolapio, 99	Savary-Gilliard (88) *Dilation with bougies up to 17 mm*	Bard (82) + Microvasive (81) *Dilation with balloons up to 15 mm*	Peptic stricture (114) Schatzki ring (139)

n/a: not applicable.
